# Coarse-Grained Quantum Theory of Organic Photovoltaic Devices

**DOI:** 10.3390/nano11020495

**Published:** 2021-02-16

**Authors:** Fernando Sánchez, Vicenta Sánchez, Chumin Wang

**Affiliations:** 1 Instituto de Investigaciones en Materiales, Universidad Nacional Autónoma de México, Mexico City 04510, Mexico; f.sanchez@ciencias.unam.mx; 2 Departamento de Física, Facultad de Ciencias, Universidad Nacional Autónoma de México, Mexico City 04510, Mexico; vicenta@unam.mx

**Keywords:** bulk heterojunction, exciton dissociation, quantum efficiency, short-circuit current, effective channel method

## Abstract

Understanding the exciton dissociation process in organic solar cells is a fundamental issue for the design of high-performance photovoltaic devices. In this article, a parameterized quantum theory based on a coarse-grained tight-binding model plus non-local electron-hole interactions is presented, while the diffusion and recombination of excitons are studied in a square lattice of excitonic states, where a real-space renormalization method on effective chains has been used. The Hamiltonian parameters are determined by fitting the measured quantum efficiency spectra and the theoretical short-circuit currents without adjustable parameters show a good agreement with the experimental ones obtained from several polymer:fullerene and polymer:polymer heterojunctions. Moreover, the present study reveals the degree of polymerization and the true driving force at donor-acceptor interface in each analyzed organic photovoltaic device.

## 1. Introduction

Harvesting solar energy through photovoltaic devices constitutes one of the most active ways to solve the rapid growth of global electricity requirements, given that in one hour the earth receives enough energy from the sun to satisfy current human needs for a year [1]. In fact, the solar cells are generally of low maintenance and may directly convert the abundant sunlight into electric power without emitting gas and noise. Recently, organic photocells have received extensive attention because of their exceptional advantages, such as their light weight, low cost, mechanical flexibility, high transparency, and roll-to-roll large area processing [2]. Nevertheless, its current power conversion efficiency (PCE) is still far below that obtained in inorganic solar cells [3].

In organic photovoltaic devices, the bulk heterojunction active layer is built by a blend of electron donors, such as P3HT [4] or PM6 [5], and acceptors like PC_71_BM [6] or nanostructured material Y6 [7], forming an interpenetrating coarse-grained molecular network [8] whose donor-acceptor interfacial area plays a crucial role for the exciton diffusion and device PCE [9]. There is a driving force at such a donor-acceptor interface originated from the electron-affinity difference between donor and acceptor molecules. This driving force may overcome the electron-hole attractive Coulomb interaction and yields an enhanced charge separation in the active layer to be collected by the cathode and anode contacts [10].

The first organic solar cell was built by C.W. Tang and S.A. VanSlyke in 1986 and contains a double layer of organic thin films as photoactive cell with an external quantum efficiency (EQE) of 1% [11]. In 1995, A. J. Heeger and collaborators introduced the concept of bulk heterojunction structures based on a network of donor-acceptor heterojunctions made of MEH-PPV:C_60_ composite and reported a PCE of 2.9% [12]. Since then, there is a continuous improvement of the PCE of organic solar cells by varying chemical and nano structural compositions of the active layer [13] and very recently a PCE of 18% has been obtained [14].

The efficiency of an organic photovoltaic device is sensitively dependent on the active layer morphology, whose structure can be divided into three sections in a polymer:fullerene bulk heterojunction: (1) a pure polymer region, (2) a pure fullerene region, and (3) donor:acceptor interface one [15]. The latter has a crucial role in the light absorption, electron-hole recombination, and exciton dissociation, and therefore determines the PCE. First principle approaches, such as the time-dependent density functional theory, have been used to calculate the excited electronic states of a wide range oligomer:fullerene [16], polymer:fullerene [17], and polymer:non-fullerene [18] composites. The formation of a complex interface morphology was studied by using ab-initio molecular dynamics [19].

In general, a coarse-grained quantum mechanical approach lies between purely empirical and totally ab initio methods [20]. The former summarizes experimental data via mathematical tools, for example polynomial fitting, while the latter attempts to explain natural phenomena from only first principles without the use of any observational information. In contrast, the semiempirical strategy develops the theory from first principles with meaningful parameters determined by the measurements. In fact, the semi-empirical coarse-grained quantum models have the advantages of being conceptually clear, computationally efficient without self-consistent processes, and able to investigate the long-range exciton diffusion in complex organic composites, as well as being able to calculate measurable device properties, such as the internal quantum efficiency (IQE) and the short-circuit current ($J_{SC}$).

In this article, we present a quantum theory based on an attractive coarse-grained Hubbard model for a pair of electron and hole, named as exciton. The excitonic states in this theory can be arranged as a square lattice with impurity sites originated from the local and non-local electron-hole attractive interactions when the scape leads to the cathode and anode, respectively for electrons and holes, are modeled by linear chains of coarse-grained molecules. For an accurate study of the exciton diffusion in organic solar cells, large lattices of excitonic states should be considered and an efficient way to address such lattices could be the real-space renormalization plus effective chain method [21], as well as its extension developed in Appendix A to include the non-local electron-hole interactions. The calculated IQE and $J_{SC}$ are compared with those measured in several polymer: fullerene and polymer: non-fullerene photovoltaic devices.

## 2. The Model

An organic photovoltaic device based on bulk heterojunctions is built by a blend of two organic materials in its active layer: one works as an electron donor and the other as an electron acceptor. Such an active layer is inserted in between the cathode and anode electrical contacts, which collect the dissociated electron and hole charge carriers from the donor-acceptor interface.

The performance of a photovoltaic device is restricted by the recombination of photoexcited excitons, which can be reduced by improving the excitonic diffusion and dissociation. However, the electron-hole Coulomb attraction inhibits such dissociation and a proper choice of donor and acceptor molecules in organic solar cells creates a driving force to overcome the mentioned attraction. Moreover, the diffusion of electrons and of holes along their scape leads plays another crucial element in the design of high performance organic photovoltaic devices.

To model the diffusion, dissociation, and recombination of excitons, we consider a coarse-grained Hubbard Hamiltonian with local (U), non-local first-neighbor (V), and second-neighbor (W) electron-hole interactions [22] around the molecular photocell labeled as site zero, which is connected to two long coarse-grained one-dimensional (1D) leads. Such a Hamiltonian can be written as

(1) $$\begin{array}{l} {\hat{H}}_{e-h}\;=\sum_{l=0}^{N_{e}}\varepsilon_{l}^{e}\;{\hat{e}}_{l}^{†}\;{\hat{e}}_{l}+\sum_{l=0}^{N_{h}}\varepsilon_{l}^{h}\;{\hat{h}}_{l}^{†}\;{\hat{h}}_{l}+\overset{N_{e}}{\underset{\underset{\mathrm{with}\;\langle l,j\rangle }{l,j=0}}{\sum }}t_{l,j}^{e}\;{\hat{e}}_{l}^{†}\;{\hat{e}}_{j}+\overset{N_{h}}{\underset{\underset{\mathrm{with}\;\langle l,j\rangle }{l,j=0}}{\sum }}t_{l,j}^{h}\;{\hat{h}}_{l}^{†}\;{\hat{h}}_{j}\\ +U\;{\hat{e}}_{0}^{†}\;{\hat{e}}_{0}\;{\hat{h}}_{0}^{†}\;{\hat{h}}_{0}+V({\hat{e}}_{0}^{†}\;{\hat{e}}_{0}\;{\hat{h}}_{1}^{†}\;{\hat{h}}_{1}\;+{\hat{e}}_{1}^{†}\;{\hat{e}}_{1}\;{\hat{h}}_{0}^{†}\;{\hat{h}}_{0})+W\;{\hat{e}}_{1}^{†}\;{\hat{e}}_{1}\;{\hat{h}}_{1}^{†}\;{\hat{h}}_{1}, \end{array}$$

where $\langle l,j\rangle$ indicates the nearest neighbor molecules, while ${\hat{e}}_{l}^{†}({\hat{e}}_{l})$ and ${\hat{h}}_{l}^{†}({\hat{h}}_{l})$ are respectively the creation (annihilation) operators of electrons and holes at molecule *l* in both leads of $N_{e}$ and $N_{h}$ coarse-grained molecules correspondingly with self-energies $\varepsilon_{l}^{e}$ and $\varepsilon_{l}^{h}=0$. In Equation (1), $t_{l,j}^{e}$ ($t_{l,j}^{h}$) are the hopping integrals of electron (hole) in their respective leads connecting to cathode (anode) contacts, where $t_{l,j}^{e}=t_{l,j}^{h}=t$ except when $t_{0,1}^{e}=t_{1,0}^{e}=t_{e}$ and $t_{0,1}^{h}=t_{1,0}^{h}=t_{h}$.

**Figure 1:**
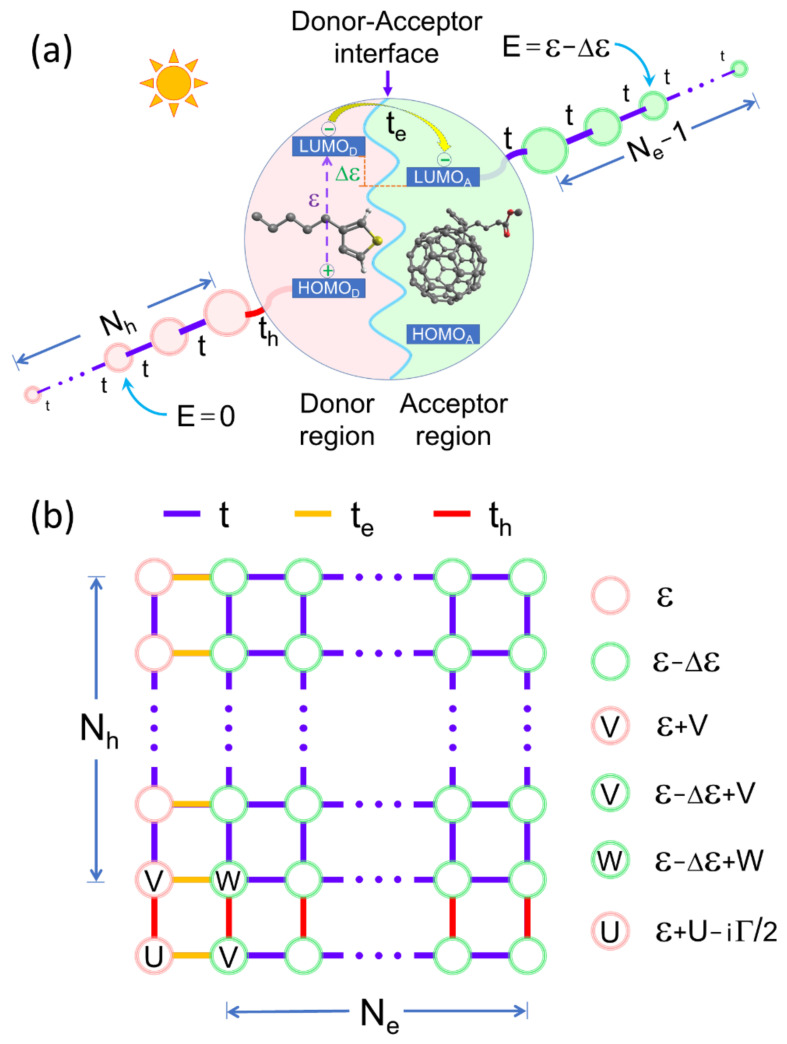
(Color online) (**a**) Schematic representation of a donor-acceptor interface, whose photocell has connections to two leads of *N_e_* coarse-grained molecules for electrons and of *N_h_* ones for holes. (**b**) The square lattice of excitonic states originated from a Hamiltonian (1) with local (U) and non-local (V, W) electron-hole interactions represented by impurity sites.

Figure 1a shows a schematic sketch of the donor-acceptor interface in an organic solar cell, where Poly(3-hexylthiophene-2.5-diyl) or P3HT works as the electron donor while [6,6]-phenyl-C_61_-butyric acid methyl ester or PC_61_BM acts as the electron acceptor. The highest occupied and lowest unoccupied molecular orbital energies of donor are represented by HOMO_D_ and LUMO_D_, while those of acceptor are respectively denoted by HOMO_A_ and LUMO_A_. The purple dashed arrow indicates the electron excitation from the HOMO_D_ to LUMO_D_ by absorbing a photon and leaves a hole at the HOMO_D_. Before the photoexcited electron transfers to the acceptor side, these electron-hole pairs may recombine with a probability of Γ emitting a new photon [23,24].

The energy difference between LUMO_D_ and LUMO_A_ gives rise to a driving force (Δε), which favors the electron transfer to the acceptor side across the donor-acceptor interface. Once the electron is found at the acceptor side while the hole still stays at the donor one, these charge carriers may move to the cathode and anode through two leads with the hopping integral t of $N_{e}$ coarse-grained molecules for electrons and of $N_{h}$ ones for holes. The connection between the donor molecule (photocell) and the first molecule of escape lead for electrons and that for holes are respectively characterized by the hopping integrals $t_{e}$ and $t_{h}$, as shown in Figure 1a.

The excitonic states with self-energy $\varepsilon_{l}^{e}+\varepsilon_{l}^{h}$, resulting from the Hamiltonian (1), can be arranged as a rectangular lattice of $\left(N_{e}\;+1)\times (N_{h}\;+1\right)$ sites shown in Figure 1b, similar to that of electron pairs [25]. For example, the left column (bottom row) in Figure 1b describes the hole (electron) movement along the escape lead, while its partner electron (hole) stays at the donor molecule.

The IQE of organic photovoltaic devices can be calculated in terms of the electron-hole recombination rate (Γ) and the effective self-energy at the impurity site U, $\Sigma_{0}\;(E)$, when both the electron and the hole are found at the photocell or donor molecule. In this article, $\Sigma_{0}\;(E)$ is determined by renormalizing the rest excitonic states of Figure 1b, as shown in Appendix A, and then contains the participation of whole excitonic lattice. In general, IQE can be written as [21,23]

(2) IQE(E)=−ImΣ0 ( E )Γ/2−ImΣ0 ( E ),

where E is the energy of the photon or that of the exciton if the energy of HOMO_D_ is placed at zero. Moreover, $J_{SC}$ can be calculated by means of [26]

(3) $$J_{SC}=\frac{e}{h\;c}\int \lambda \;\Phi (\lambda )\;EQE(\lambda )\;d\lambda =e\;\;h\;c\;\int \frac{1}{E^{3}}\Phi (E)\;EQE(E)\;dE,$$

where h is the Planck constant, *e* is the electrical charge of electron, *c* is the speed of light in a vacuum, λ=hc/E is the photon wavelength, Φ(λ) is the AM1.5G solar spectrum, and EQE(λ) is related to IQE(λ) through [27,28]

(4) EQE(λ)=IQE(λ)×Abs(λ),

with Abs(λ) being the absorption spectrum of organic solar cells.

## 3. Numerical and Analytical Results

**Figure 2:**
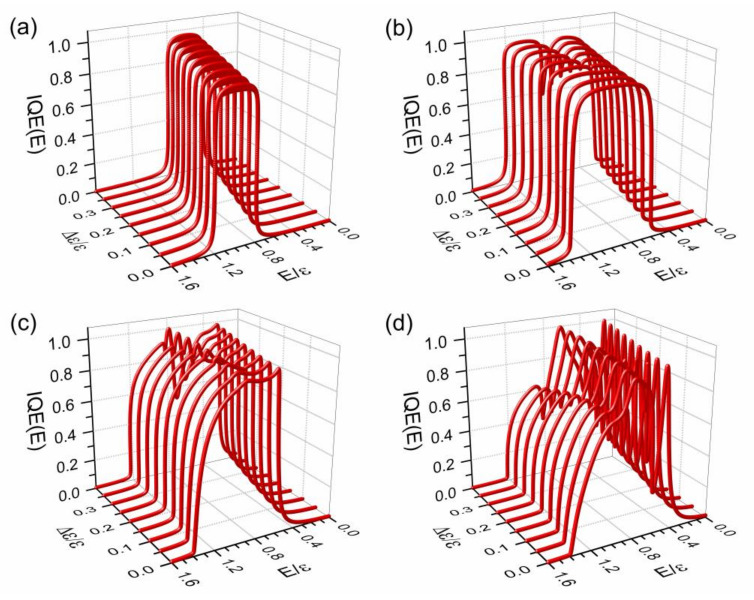
(Color online) Internal quantum efficiency (IQE) as a function of the driving force (Δε ) and photon energy (E) for a lattice of $(N_{h}+1)\times (N_{e}+1)=\;67,108,864\;\times \;200$ excitonic states with arbitrary local Coulomb interaction U, an electron-hole recombination rate Γ=0.01ε and hoping integrals $t=t_{e}=t_{h}=-\;0.1\varepsilon$ except in (**a**) for $t_{h}=0$ on a reduced excitonic lattice of $N_{e}\;+1=200$ states. The used non-local electron-hole interactions are (**a**,**b**) V=W=0, (**c**) V=2W=− 0.2ε, and (**d**) V =2 W =−0.4ε.

The performance of an organic solar cell can be quantified through IQE as defined in Equation (2), as well as $J_{SC}$ provided by Equation (3). In Figure 2, IQE is plotted as a function of the driving force (Δε) and photon energy (*E*) for an excitonic lattice of $(N_{h}+1)\times (N_{e}+1)=\;67,108,864\;\times \;200$ states with (a) $t_{h}\;=V\;=W\;=0$ and (b–d) $t_{h}=-\;0.1\varepsilon$, beside when (b) V =W =0, (c) V =2W =− 0.2ε, and (d) V =2W =− 0.4ε. Also, an electron-hole recombination rate Γ=0.01ε, hopping integrals $t_{e}\;=t=-\;0.1\varepsilon$, an imaginary part of the energy $\eta =10^{-3}\varepsilon$ and arbitrary local Coulomb interaction strength (U) are used in Figure 2. The numerical calculation was carried out by means of a real-space renormalization method [29] on independent chains illustrated in Appendix A.

For the case of $t_{h}\;=0$, the excitonic lattice becomes to a single chain at the bottom of Figure 1b and its $\Sigma_{0}\;(\;E\;)$ at the impurity site U, numbered as the zero site, has an analytical solution given by $\Sigma_{0}(E)=t_{e}^{2}\left[(E-\varepsilon +\Delta \varepsilon )-\sqrt{{\left(E-\varepsilon +\Delta \varepsilon \right)}^{2}\;-4t^{2}}\right]/2\;t^{2}$, as obtained in Appendix B. As a consequence, Equation (2) converts to

(5) $$IQE(E)=\left\{\begin{array}{cc} \frac{t_{e}^{2}\sqrt{4t^{2}-{\left(E-\varepsilon +\Delta \varepsilon \right)}^{2}}}{\Gamma t^{2}\;+t_{e}^{2}\sqrt{4t^{2}-{\left(E-\varepsilon +\Delta \varepsilon \right)}^{2}}}, & \;\mathrm{if}\;\varepsilon -\Delta \varepsilon -2|t|\;<E<\varepsilon -\Delta \varepsilon +2|t|\\ 0, & \;\mathrm{other}\;\mathrm{cases}\; \end{array}\right.$$

which reproduces the numerical results shown in Figure 2a, except that the numerical ones contain an imaginary part of the energy $\eta =10^{-3}\varepsilon$ and were carried out on a finite chain of 200 excitonic states in contrast to a semi-infinite chain considered in the analytical study.

In Figure 2b, an IQE band splitting is observed as Δε grows, where the band at high energy region is produced by the left column of $N_{h}+1$ excitonic states mainly with self-energy ε, red circles in Figure 1b, while that of the low energy region is originated from the rest of the excitonic states in Figure 1b with a self-energy ε−Δε (green circles) [30]. Moreover, when non-local electron-hole Coulomb attractions are considered, sharp peaks appear at the low energy region in Figure 2d, due to the presence of impurity sites in the square lattice of excitonic states, as seen in Figure 1b.

**Figure 3:**
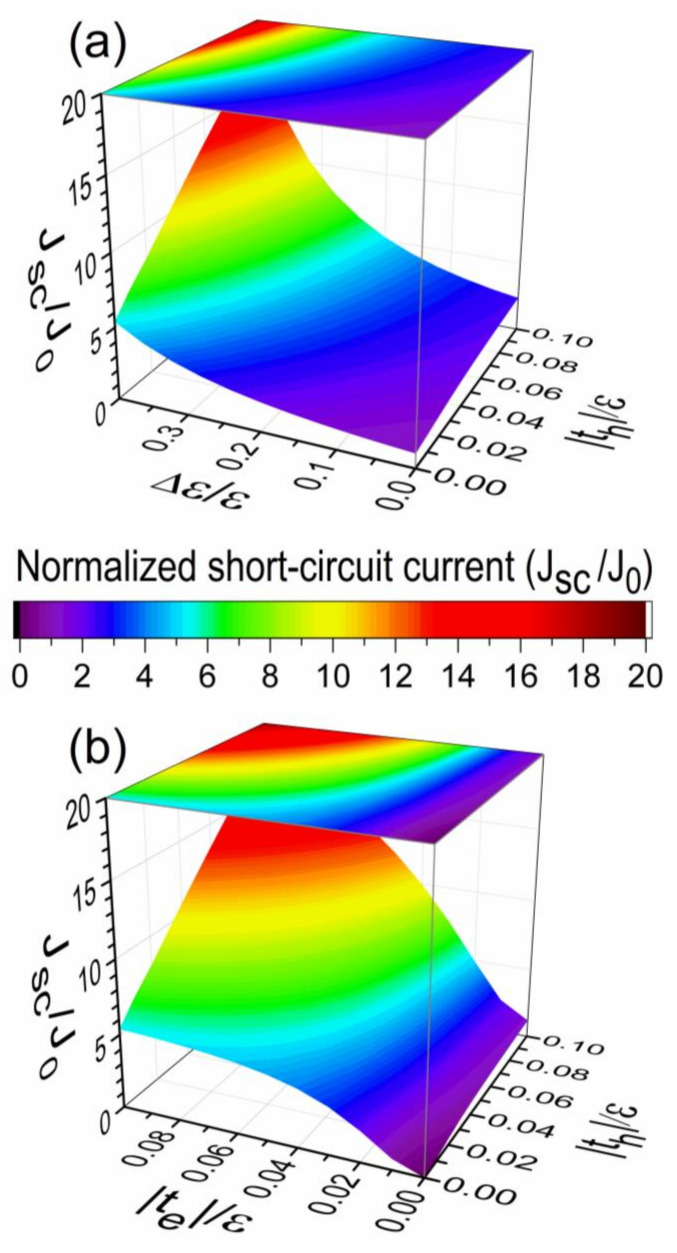
(Color online) Normalized short-circuit current ($J_{SC}/J_{0}$) (**a**) as a function of the hopping integral $t_{h}$ and Δε with $t_{e}=-\;0.1\varepsilon$ and (**b**) versus hopping integrals $t_{e}$ and $t_{h}$ with Δε=0.4ε for an excitonic lattice of $(N_{h}+1)\times (N_{e}+1)=\;67,108,864\;\times \;200$ states. The electron-hole recombination rate used was Γ=0.01 ε and an imaginary part of energy of $\eta =10^{-3}\varepsilon$.

In Figure 3, the normalized short-circuit current ($J_{SC}/J_{0}$) is plotted (a) as a function of the hopping integral $t_{h}$ and the driving force Δε with $t_{e}=-\;0.1\varepsilon$, while (b) versus hopping integrals $t_{h}$ and $t_{e}$ with Δε=0.4ε for the same excitonic lattice parameters used in Figure 2b. The normalization current $\left(J_{0}\right)$ is given by $J_{0}=J_{SC}(t_{h}=0,\Delta \varepsilon =0)$ with V=W=0, $t_{e}=t=-\;0.1\varepsilon$, Γ=0.01ε, and an arbitrary U, whose analytical expression is given in Appendix B. It is possible to observe the growth of $J_{SC}/J_{0}$ from its minimum value of one in Figure 3a when $t_{h}$ or Δε increases, where the raise of $\left|t_{h}\right|$ from zero to 0.1ε becomes the lattice of excitonic states from 1D into a two-dimensional (2D) system while Δε enlarges the IQE bandwidth by splitting such a band, as shown in Figure 2b.

In Figure 3b, it is possible to observe the increase of $J_{SC}$ from its minimum value of zero when $t_{e}$ and $t_{h}$ increase, given that for $t_{h}=t_{e}=0$, the photocell is disconnected from its leads and then $J_{SC}=0$. Also, note the faster growth of $J_{SC}$ with $t_{e}$ than its increase with $t_{h}$, because $t_{e}$ adds the participation of driving force as shown in Figure 1b, and a symmetrical growth of $J_{SC}$ with both $t_{e}$ and $t_{h}$ would be observed if Δε=0. Finally, the curves of $J_{SC}$ presented in Figure 3a,b for $t_{h}=0$ can be confirmed by the analytical results given in Appendix B.

## 4. Theory Versus Experiments

**Figure 4:**
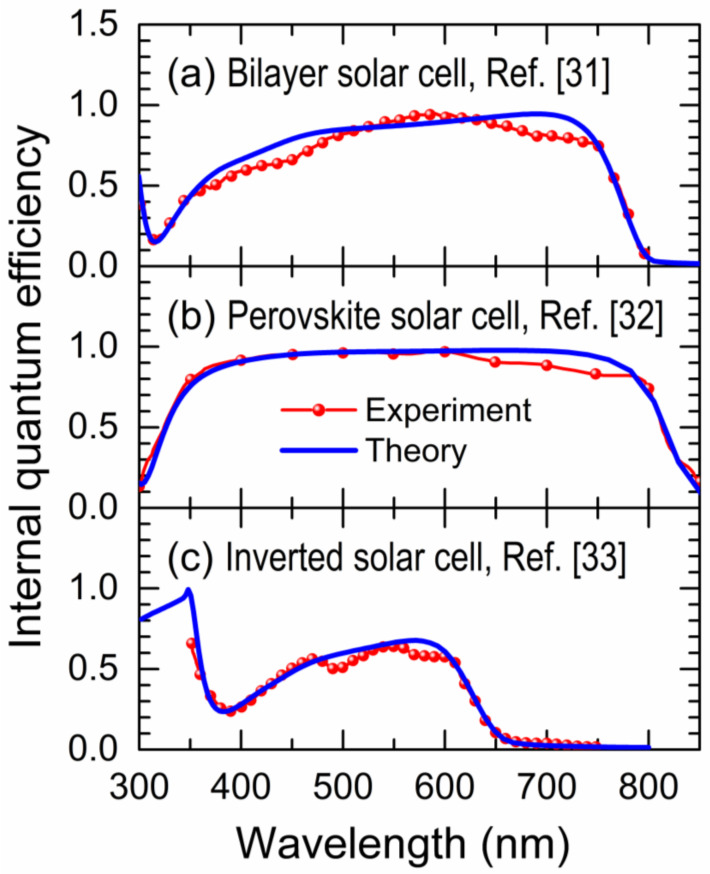
(Color online) Experimental (red spheres) and theoretical (blue line) internal quantum efficiency (IQE) versus photon wavelength (*λ*) for (**a**) bilayer [31], (**b**) perovskite [32], and (**c**) inverted [33] solar cells.

In this section, we use the present coarse-grained quantum theory to model diverse organic photovoltaic devices, such as those of a bilayer in Figure 4a, perovskite in Figure 4b, and inverted solar cells in Figure 4c, whose reported IQE (red spheres) [31,32,33] are compared with the theoretical ones (blue lines).

Note in Figure 4 that the theoretical IQE spectra are able to reproduce the main features of the experimental ones. The parameters used for each organic solar cell are summarized in Table 1, where the difference between the HOMO_D_ and LUMO_D_ energies (ε) at the photocell or donor molecule depends on, for example, the amount of monomer in the P3HT polymer when it acts as the photocell [34,35].

The corresponding short-circuit currents ($J_{SC}$) have been calculated by means of Equation (3), in which the theoretical IQE spectra of Figure 4a–c, the measured photon absorption spectrum of each solar cell, and the standard AM1.5G solar spectrum data (ASTM G-173-03) [36] were used. The results of calculated $J_{SC}$ are shown in Table 2 and compared with those reported in Refs. [31,32,33]. Note the remarkable agreement between the measured $J_{SC}$ and the theoretical ones without adjustable parameters, obtaining a maximum difference of 5.30%.

## 5. Conclusions

In this article, the diffusion, dissociation, and recombination of light-induced excitons in organic photovoltaic devices with 1D coarse-grained molecular leads for electrons and for holes have been investigated by means of a 2D lattice of excitonic states with impurities that are originated from the local (U) and non-local (V, W) electron-hole Coulomb interactions. The internal quantum efficiency (IQE) and short-circuit current ($J_{SC}$) were calculated by using an independent chain method explained in Appendix A. The results reveal a crucial role of the driving force (Δε) generated by the difference between the LUMO_D_ and LUMO_A_ energies in both IQE and $J_{SC}$, since it splits and widens the excitonic band. For the case of null hopping integral $t_{h}$, the numerical results were verified by the analytical ones presented in Appendix B.

The semiempirical Hamiltonian parameters, such as the LUMO-HOMO energy difference at photocell (ε), driving force (Δε) and hopping integrals (*t*, $t_{e}$, and $t_{h}$) were determined for diverse organic photovoltaic devices by fitting their IQE spectra. The calculated $J_{SC}$ without adjustable parameters have been compared with the experimental data and a good agreement with a maximal deviation less than 6% was obtained for the three different-type analyzed photovoltaic devices [31,32,33]. Finally, we think the presented coarse-grained quantum theory has the merit of being conceptually clear, mathematically simple, computationally efficient, and widely versatile to investigate the long-range exciton diffusion in complex organic composites, as well as to calculate measurable device properties. In fact, it captures the main features of organic solar cells and may contribute to understanding the exciton dissociation-recombination competition, as well as to obtaining information about their microscopic structures; for example, the determined ε could be related to the polymerization degree of photocell in each analyzed organic photovoltaic device.

## Figures and Tables

**Table 1:** Semiempirical parameters in eV used in Figure 4 with $t_{h}=t$, W=V/2 and arbitrary *U.*

Solar Cell	ε	Δ*ε*	Γ	*t*	$t_{e}$	V	*η*	Ref.
ITO/PEDOT:PSS/ CH_3_NH_3_Pbl_3_/PC_61_BM/Al	5.30	2.01	0.05	−0.437	−0.30	−0.83	$10^{-3}$	[31]
FAPbl3 perovskite	5.28	2.25	0.01	−0.393	−0.272	−0.55	$10^{-3}$	[32]
ITO/SnO2/PEIE/ P3HT:PCBM/MoO3/Ag	4.07	1.15	0.05	−0.264	−0.115	−0.35	$9.9\times 10^{-3}$	[33]

**Table 2:** Comparison of theoretical and experimental $J_{SC}$.

Solar Cell	$J_{SC}^{Theory}\;(mA/cm^{2})$	$J_{SC}^{Exp}\;(mA/cm^{2})$	Error (%)	Reference
ITO/PEDOT:PSS/CH_3_NH_3_Pbl_3_/PC_61_BM/Al	8.526	8.201	3.96	[31]
FAPbl3 perovskite	20.72	21.88	5.30	[32]
ITO/SnO2/PEIE/P3HT:PCBM/MoO3/Ag	8.26	7.86	5.08	[33]

**Figure A1:**
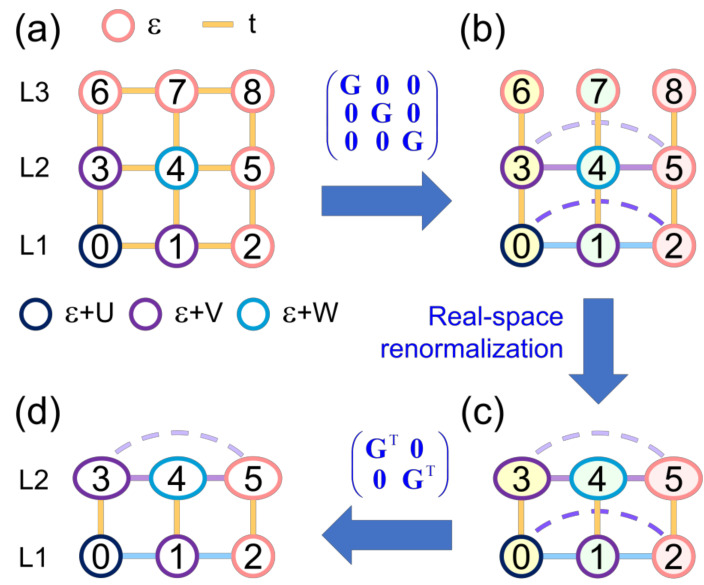
(Color online) (**a**) Square lattice of 3×3 excitonic states with local (U) and non-local (V, W) electron-hole interactions of Hamiltonian (1); (**b**) After applying a unitary transformation, appear three independent chains 3–6, 4–7 and 5–8, as well as new hopping connections on L1 and L2 levels; (**c**) Via a renormalization procedure, sites 6, 7 and 8 were eliminated but their participation is preserved through the new self-energies of sites 3, 4 and 5 illustrated as ellipses; (**d**) Final excitonic lattice after an inverse unitary transformation, where the sites on L2 remain totally interconnected while those on L1 restore to their original structure.

## Data Availability

Data sharing not applicable.

## Appendix A. Effective Chain Method

The square lattice of excitonic states, showed in Figure 1b, contains impurity sites originated from local (U) and non-local (V, W) interactions of Hamiltonian (1) and can be studied by means of the effective channel method developed in Ref. [21], in which by means of a unitary transformation the 2D excitonic lattice is converted into a set of 1D effective chains interconnected only at one of their ends. For the case of V≠0 and/or W≠0, the mentioned method should be extended to that shown in Figure A1 for a square lattice of 3×3 excitonic states.

For a square lattice of 3 × 3 excitonic states, Hamiltonian (1) can be written, following the numbering of Figure A1a, as

(A1) $$H=\left(\begin{array}{ccc} \Xi (U,V) & \Lambda & 0\\ \Lambda & \Xi (V,W) & \Lambda \\ 0 & \Lambda & \Xi (0,0) \end{array}\right),$$

where $\Xi (\alpha ,\beta )=\left(\begin{array}{ccc} \varepsilon +\alpha & t & 0\\ t & \varepsilon +\beta & t\\ 0 & t & \varepsilon \end{array}\right)$ and $\Lambda =\left(\begin{array}{ccc} t & 0 & 0\\ 0 & t & 0\\ 0 & 0 & t \end{array}\right)$, being α=U, V or 0 while β=V, W or 0.

A new Hamiltonian ($\tilde{H}$) corresponding to the lattice shown in Figure A1b can be obtained by applying the following unitary transformation to **H**,

(A2) $$\tilde{H}=\left(\begin{array}{ccc} G^{T} & 0 & 0\\ 0 & G^{T} & 0\\ 0 & 0 & G^{T} \end{array}\right)\left(\begin{array}{ccc} \Xi (U,V) & \Lambda & 0\\ \Lambda & \Xi (V,W) & \Lambda \\ 0 & \Lambda & \Xi (0,0) \end{array}\right)\left(\begin{array}{ccc} G & 0 & 0\\ 0 & G & 0\\ 0 & 0 & G \end{array}\right)=\left(\begin{array}{ccc} \tilde{\Xi }(U,V) & \Lambda & 0\\ \Lambda & \tilde{\Xi }(V,W) & \Lambda \\ 0 & \Lambda & \tilde{\Xi }(0,0) \end{array}\right),$$

where $G=\left(\begin{array}{ccc} \frac{1}{2} & \frac{-1}{\sqrt{2}} & \frac{1}{2}\\ \frac{1}{\sqrt{2}} & 0 & \frac{-1}{\sqrt{2}}\\ \frac{1}{2} & \frac{1}{\sqrt{2}} & \frac{1}{2} \end{array}\right)$ and $\tilde{\Xi }(\alpha ,\beta )=\left(\begin{array}{ccc} \frac{1}{4}\alpha +\frac{1}{2}\beta +\varepsilon +\sqrt{2}t & -\frac{1}{2\sqrt{2}}\alpha & \frac{1}{4}\alpha -\frac{1}{2}\beta \\ -\frac{1}{2\sqrt{2}}\alpha & \frac{1}{2}\alpha +\varepsilon & -\frac{1}{2\sqrt{2}}\alpha \\ \frac{1}{4}\alpha -\frac{1}{2}\beta & -\frac{1}{2\sqrt{2}}\alpha & \frac{1}{4}\alpha +\frac{1}{2}\beta +\varepsilon -\sqrt{2}t \end{array}\right)$. Notice that $\tilde{\Xi }(0,0)$ has null off-diagonal elements since α=β=0, i.e., the hopping integrals between sites 6, 7 and 8 are zero as illustrated in Figure A1b, while the sites on L1 and L2 levels are now totally interconnected as respectively shown by $\tilde{\Xi }(U,V)$ and $\tilde{\Xi }(V,W)$.

If a real-space renormalization or decimation of sites 6, 7 and 8 is performed, the self-energies of sites 3, 4 and 5 are modified, shown as ellipses in Figure A1c, which converts $\tilde{\Xi }(V,W)$ into

(A3) $$\overset{⌢}{\Xi }(V,W)=\left(\begin{array}{ccc} \frac{V}{4}+\frac{W}{2}+\varepsilon +\sqrt{2}t+\frac{t^{2}}{E-\varepsilon -\sqrt{2}t} & -\frac{V}{2\sqrt{2}} & \frac{V}{4}-\frac{W}{2}\\ -\frac{V}{2\sqrt{2}} & \frac{V}{2}+\varepsilon +\frac{t^{2}}{E-\varepsilon } & -\frac{V}{2\sqrt{2}}\\ \frac{V}{4}-\frac{W}{2} & -\frac{V}{2\sqrt{2}} & \frac{V}{4}+\frac{W}{2}+\varepsilon -\sqrt{2}t+\frac{t^{2}}{E-\varepsilon +\sqrt{2}t} \end{array}\right).$$

Hence, the reduced excitonic lattice of 6 sites, Figure A1c, is described by Hamiltonian ($\overset{⌢}{H}$) given by

(A4) $$\overset{⌢}{H}=\left(\begin{array}{cc} \tilde{\Xi }(U,V) & \Lambda \\ \Lambda & \overset{⌢}{\Xi }(V,W) \end{array}\right).$$

Finally, applying an inverse unitary transformation to $\overset{⌢}{H}$, we obtain a reduced Hamiltonian ($H_{R}$) given by

(A5) $$H_{R}=\left(\begin{array}{cc} G & 0\\ 0 & G \end{array}\right)\left(\begin{array}{cc} \tilde{\Xi }(U,V) & \Lambda \\ \Lambda & \overset{⌢}{\Xi }(V,W) \end{array}\right)\left(\begin{array}{cc} G^{T} & 0\\ 0 & G^{T} \end{array}\right)=\left(\begin{array}{cc} \Xi (U,V) & \Lambda \\ \Lambda & \overset{⌣}{\Xi }(V,W) \end{array}\right),$$

where,

(A6) $$\overset{⌣}{\Xi }(V,W)=\left(\begin{array}{ccc} \varepsilon +V+\frac{\kappa \;t^{2}}{E-\varepsilon } & \kappa \;t & \frac{t^{4}{\left(E-\varepsilon \right)}^{-1}}{{\left(E-\varepsilon \right)}^{2}-2t^{2}}\\ \kappa \;t & \varepsilon +W+\frac{t^{2}(E-\varepsilon )}{{\left(E-\varepsilon \right)}^{2}-2t^{2}} & \kappa \;t\\ \frac{t^{4}{\left(E-\varepsilon \right)}^{-1}}{{\left(E-\varepsilon \right)}^{2}-2t^{2}} & \kappa \;t & \varepsilon +\frac{\kappa \;t^{2}}{E-\varepsilon } \end{array}\right)$$

with $\kappa =\frac{{\left(E-\varepsilon \right)}^{2}-t^{2}}{{\left(E-\varepsilon \right)}^{2}-2t^{2}}$. Notice that the sites 1, 2 and 3 on L1 level are restored to the original structure described by Ξ(U,V), while the sites 4, 5 and 6 on L2 level remain totally interconnected determined by $\overset{⌣}{\Xi }(V,W)$.

## Appendix B. Analytical Solution of Short-Circuit Current

The excitonic states obtained from Hamiltonian (1) with V =W =0 can be arranged as a square lattice containing a unique impurity site U at its corner in Figure 1b with self-energy $\varepsilon_{U}=\varepsilon +U-i\;\Gamma /2$, when the electron and hole are located at the photocell. For the case of $t_{h}=0$, the horizontal chain in Figure 1b that contains such impurity site is disconnected from the rest of excitonic states. In consequence, the effective self-energy at such impurity site, $\Sigma_{0}\;(E)$, is given by

(A7) $$\Sigma_{0}(E)=\frac{t_{e}^{2}}{E-\varepsilon +\Delta \varepsilon -\Sigma_{1}(E)},$$

where $\Sigma_{1}(E)$ is the effective self-energy at the first neighbor of the impurity site, named as site 1, and it can be calculated by means of the continued fraction procedure for a semi-infinite chain [21]

(A8) $$\Sigma_{1}(E)=\frac{t^{2}}{E-\varepsilon +\Delta \varepsilon -\Sigma_{1}(E)},$$

which leads to ${\left[\Sigma_{1}(E)\right]}^{2}\;-(E-\varepsilon +\Delta \varepsilon )\;\Sigma_{1}(E)+t^{2}\;=0$, whose solutions are

(A9) $$\Sigma_{1}(E)=\frac{1}{2}\left[E-\varepsilon +\Delta \varepsilon \pm \sqrt{{\left(E-\varepsilon +\Delta \varepsilon \right)}^{2}-4t^{2}}\right],$$

where the plus sign and minus sign of $\Sigma_{1}(E)$ respectively provide negative and positive local density of states (LDOS) at site 1 for |E−ε+Δε|<2|t|. Substituting the minus sign solution of (A9) in (A7) we obtain

(A10) $$\Sigma_{0}(E)=\frac{t_{e}^{2}}{2t^{2}}\left[E-\varepsilon +\Delta \varepsilon -\sqrt{{\left(E-\varepsilon +\Delta \varepsilon \right)}^{2}-4t^{2}}\right].$$

Hence, the internal quantum efficiency (*IQE*) is given by

(A11) IQE(E)=−ImΣ0 (E)Γ/2−ImΣ0 ( E )=te24t2−(E−ε+Δε)2Γt2+te24t2−(E−ε+Δε)2,

when $E_{-}\;<E<E_{+}$ with $E_{\pm }\;=\varepsilon -\Delta \varepsilon \pm 2|t|$.

For the ideal case of a unitary absorption spectrum, Abs(λ)=1, and a unitary solar spectrum,  Φ(λ)=1, the external quantum efficiency (EQE) of Equation (4) leads to EQE(λ)=IQE(λ) and the short-circuit current ($J_{SC}$) of Equation (3) can be rewritten as

(A12) $$J_{SC}=\frac{e}{h\;c}\int_{\lambda_{+}}^{\lambda_{-}}\lambda \;IQE(\lambda )\;d\lambda =\frac{e}{h\;c}\int_{\lambda_{+}}^{\lambda_{-}}\lambda \;\frac{t_{e}^{2}\sqrt{4t^{2}-{\left(hc/\lambda -\varepsilon +\Delta \varepsilon \right)}^{2}}}{\Gamma t^{2}+t_{e}^{2}\sqrt{4t^{2}-{\left(hc/\lambda -\varepsilon +\Delta \varepsilon \right)}^{2}}}\;d\lambda$$

where E=hc/λ and $\lambda_{\pm }\;=hc/E_{\pm }$ have been used. The integral of Equation (A12) can be rewritten as

(A13) $$J_{SC}=\frac{e}{hc}\left[\int_{\lambda_{+}}^{\lambda_{-}}\frac{\lambda (-A\lambda^{2}+B\lambda -C)}{-D\lambda^{2}+B\lambda -C}d\lambda -\frac{t^{2}\Gamma }{t_{e}^{2}\varepsilon }\int_{\lambda_{+}}^{\lambda_{-}}\frac{\lambda^{2}\sqrt{-A\lambda^{2}+B\lambda -C}}{-D\lambda^{2}+B\lambda -C}d\lambda \right]=\frac{e}{hc}\left[I_{1}-\frac{t^{2}\Gamma }{t_{e}^{2}\varepsilon }I_{2}\right],$$

where $A=[{\left(1-\Delta \varepsilon /\varepsilon \right)}^{2}-{\left(2t/\varepsilon \right)}^{2}]$, B=2hc(1−Δε/ε)/ε, $C={\left(hc/\varepsilon \right)}^{2}$, $D=A+{\left(t^{2}\Gamma /t_{e}^{2}\varepsilon \right)}^{2}$,

(A14) $$\begin{array}{l} I_{1}\;=\int_{\lambda_{+}}^{\lambda_{-}}\frac{\lambda (-A\lambda^{2}+B\lambda -C)}{-D\lambda^{2}+B\lambda -C}d\lambda =\frac{1}{D^{3}}\left\{(A\;-D)(B^{2}\;-CD)\ln\frac{E_{+}}{E_{-}}+2\frac{BD|t/\varepsilon |}{E_{+}^{2}\;E_{-}^{2}}\frac{hc}{\varepsilon }\left[\;AD\;+\;2E_{+}E_{-}(A\;-D)\right]\right.\\ \left.+\left[B(A\;-D)(B^{2}\;-3CD)/\sqrt{4CD-\;B^{2}}\right]\tan^{-1}\left[2|t|\sqrt{4CD-\;B^{2}}/\left(hc(D\;-A\;-8|t/\varepsilon {|}^{2})\right)\right]\;\right\} \end{array}$$

and

(A15) $$I_{2}=\int_{\lambda_{+}}^{\lambda_{-}}\frac{\lambda^{2}\sqrt{-A\lambda^{2}+B\lambda -C}}{-D\lambda^{2}+B\lambda -C}d\lambda =-\frac{\pi }{8A^{3/2}D^{3}}\left[4A(D\;-2A)(B^{2}\;-CD)+\;B^{2}D^{2}\right],$$

when $\left|t_{e}\right|\;>\sqrt{|t|\Gamma /\left(2\varepsilon \right)}$.

The limiting curves of $J_{SC}(\Delta \varepsilon )/J_{0}$ and $J_{SC}(t_{e})/J_{0}$ for $t_{h}=0$, respectively presented in Figure 3a,b, can be analytically confirmed by Equation (A13) taking $t_{e}=t=-\;0.1\varepsilon$ and Γ=0.01ε for the case of $J_{SC}(\Delta \varepsilon )/J_{0}$, while t=− 0.1ε, Γ=0.01ε and Δε=0.4ε for $J_{SC}(t_{e})/J_{0}$.

The normalization current ($J_{0}$) is given by

(A16) $$J_{0}=J_{SC}(t_{e}\;=t=-\;0.1\varepsilon ,\;t_{h}\;=0,\;\Delta \varepsilon =0,\;\Gamma =0.01\varepsilon ,\;U\;=V\;=W\;=0)\approx 0.4032383256028\;e\;h\;c/\varepsilon^{2},$$

whose numerical value was calculated from Equation (A13) and the units of $\;\Phi (\lambda )=1\;Wm^{-2}/\mathrm{nm}$ should be included in Equation (A16) to obtain the correct units of $J_{0}$.
